# Gas-Filled Phospholipid Nanoparticles Conjugated with Gadolinium Play a Role as a Potential Theragnostics for MR-Guided HIFU Ablation

**DOI:** 10.1371/journal.pone.0034333

**Published:** 2012-03-29

**Authors:** Se-Young Choi, Young-Sun Kim, Yeong-Ju Seo, Jehoon Yang, Kyu-Sil Choi

**Affiliations:** 1 Molecular and Cellular Imaging Center, Samsung Biomedical Research Institute, Sungkyunkwan University School of Medicine, Kangnam-gu, Seoul, South Korea; 2 Department of Radiology and Center for Imaging Science, Sungkyunkwan University School of Medicine, Kangnam-gu, Seoul, South Korea; National Institute of Health, United States of America

## Abstract

To develop a long-circulating theragnostics, meaning therapeutics and diagnostics for MR-guided HIFU ablation, we designed and prepared Gd-C_5_F_12_-phospholipid nanobubbles (PLNs) 30–100 nm in diameter. The biochemical and physical characterization of Gd-C_5_F_12_-PLNs were performed. Since Gd-C_5_F_12_-PLN-50 (Φ = 50 nm) and Gd-C_5_F_12_-PLN-100 (Φ = 100 nm) enhanced the hyperthermal effect of HIFU size- and concentration-dependently in a tissue-mimicking phantom, its circulation, distribution, tumor accumulation and tumor ablation were examined in tumor-bearing mice. The plasma-half life of Gd-C_5_F_12_-PLNs was longer than 1.5 hrs. Gd-C_5_F_12_-PLNs mainly accumulated in the liver and the spleen, suggesting that they are slowly secreted through the hepatobiliary pathway. Monitored by the T1 signal intensity of MR, Gd-C_5_F_12_-PLNs accumulated in tumor tissues for 8 hours in mice. HIFU with Gd-C_5_F_12_-PLN-100 showed the increased tumor ablation area as compared with HIFU alone. The results suggest that Gd-C_5_F_12_-PLNs exhibit a potential theragnostics for MR-guided HIFU ablation.

## Introduction

Minimally or non-invasive hyperthermal ablation of tumor tissues has been used for palliative and curative purposes [1]–[4]. High-intensity focused ultrasound (HIFU) ablation is even less invasive than other minimally-invasive thermal ablation techniques, such as radiofrequency, laser, and microwave ablation. Image guidance, such as ultrasound and MRI, allows HIFU ablation to deliver high intensity ultrasound (500–10,000 W/cm^2^) precisely focused on the tumor, preventing surrounding tissues from injury and complications.

Although the concept of HIFU makes it ideal for tumor ablation, it does have some shortcomings. Because the ablation zone for a single HIFU sonication is small, HIFU can require relatively long procedures, especially for large tumors [4]. In addition, hypervascular tumors require relatively high-energy HIFU sonication, which can have adverse effects such as heating intervening normal tissues including the skin [3], [5].

Microbubbles increase heat generation under HIFU mainly by increasing inertial cavitation [6]. For example, Definity® increased the ultrasound thermal effect in tissue mimicking phantoms [7], SonoVue® enhanced HIFU ablation of rabbit hepatic VX2 tumors [8], and Levovist® enhanced HIFU ablation in rat liver tumors [9]. Microbubble agents also improved the low therapeutic efficiency of kidney HIFU in rabbit [10] and goat [11] studies, and cavitation from vaporized albumin-based nanoemulsion accelerated HIFU-mediated heating [12]. It suggests that gas-filled microbubbles (Φ = 1–5 micron) have remarkable effects on HIFU-assisted tumor ablation. However, the plasma half-life of clinical microbubble contrast agents which is 10–12 min according to manufacturer's information is too short to be used as therapeutic enhancers for HIFU treatment in patients.

Thus, we designed phospholipid nanobubbles (PLNs) 30–100 nm in diameter that circulate longer than microbubbles. The PLNs also contain perfluoropentane (C_5_F_12_) gas to enhance the thermal efficacy of HIFU therapy. The C_5_F_12_-PLNs were then conjugated to gadolinium (Gd) to be detectable by MR.

The aim of this study was to determine if Gd-C_5_F_12_-PLN could be a theragnostics for hyperthermal tumor ablation by MR-guided HIFU. Various sizes (Φ = 30, 50, 100 nm) of Gd-C_5_F_12_-PLNs were prepared and their chemical characterizations were performed. The potential efficacy of Gd-C_5_F_12_-PLNs in hyperthermal therapy with HIFU was investigated in vitro and in vivo.

## Materials and Methods

### Preparation of Gd-C_5_F_12_-PLNs

Phospholipid nanobubbles were prepared according to the manufacturer's instructions with modifications<http://www.avantilipids.com>. Briefly, 25.3 mM of 1,2-dioctadecanoyl-sn-glycero-3-phosphocholine (DSPC), 22.2 mM of 1,2-dioleoyl-sn-glycero-3-phosphoethanolamine-N-[methoxy(polyethylene glycol)-350] (ammonium salt) (DOPE-PEG350), 22.8 mM of 1,2-dimyristoyl-sn-glycero-3-phosphoethanolamine-N-diethylenetriaminepentaacetic acid (ammonium salt) (DMPE-DTPA) and 17.7 mM of 1,2-dioleoyl-sn-glycero-3-phosphoethanolamine-N-[methoxy(polyethylene glycol)-350] (ammonium salt) (DSPE-PEG350) (Avanti® POLAR LIPID, Inc., Alabaster, AL) were mixed in chloroform (Sigma-Aldrich). To make the gas-filled nanobubble, 50 µl/ml of decafluoropentane (Sigma-Aldrich, St. Louis, MO) were added to these preparations. An equal volume of methanol (MERCK, Darmstadt, Germany) was added to lipid mixture. The mixed lipid was removed under evaporation. The lipid film was then dissolved in 1 ml of PBS. The mixture was sonicated for 15 minutes at 55°C in water-bath. Tween 80 (Sigma-Aldrich) was added in 75 mg/ml and sonicated again for 15 minutes at 55°C in water bath. The mixture was then loaded into one of two pre-warmed gas-tight syringes of an Extruder (Avanti® POLAR LIPID) with 30, 50, or 100 nm (cut-off) polycarbonate membranes. The entire contents of the syringe were passed through the filter and into an opposing syringe. This process was repeated for a total of more than 11 passes. The C_5_F_12_-PLN preparation was then chelated with Gd. Free Gd was removed by filtering the reaction mixture through a centrifugal filter (10,000 Da cut-off) after adding 10 ml of PBS three times. The Gd concentration in the Gd-C_5_F_12_-PLN preparation was determined by ICP-AES (Agilent 7500A, Palo Alto, CA). The magnetic susceptometry was analyzed with a superconductive quantum interference device (SQUID). The size was also determined with DLS (Dynamic light scattering). To compare stability of Gd-C_5_F_12_-PLNs with SonoVue® (Bracco, Milan, Italy) which is mainly composed of phospholipids, 40 mM [P] of Gd-C_5_F_12_-PLN-50, Gd-C_5_F_12_-PLN-100 and 5 µg/ml of SonoVue® in prewarmed saline at 37°C were sonographed in a contrast mode with high-frequency(40 MHz) ultrasound system(Vevo2100®, VisualSonics, Tronto, Canada) for 1.5 hours. The bubble solution was placed on a temperature-controlled aluminum block at 37°C during the whole experiment. Echogenic bubbles were counted from the real-time images and its % echogenesity was calculated.

### Cytotoxicity of Gd-C_5_F_12_-PLN

The cytotoxicity of Gd-C_5_F_12_-PLN was investigated with an MTT (3-(4,5-dimethylthiazol-2-yl)-2,5-diphenyltetrazolium bromide) assay in the mouse endothelial cell line, MS-1 (CRL-2279, ATCC, Manassas, VA) which is a primary contacting cell for injected PLNs in circulation. The cell lysis RIPA (radioimmunoprecipitation assay) buffer was used as a control.

### Phosphorus assay

To determine the phosphorus concentration which is the major component of Gd-C_5_F_12_-PLN, a phosphorus assay was performed. Up to 50 µl of the sample to be analyzed and 0.45 ml of 8.9 N H_2_S0_4_ (Sigma-Aldrich) were placed in a 15 mL conical centrifuge tube and heated in a 150°C oven for at least 1 hour. Then, 150 µl H_2_O_2_ was added and the solution was returned to the oven for at least 30 min more to complete the combustion and decompose all the peroxide. To the reaction tubes, 0.5 ml ammonium molybdate tetra hydrate (Sigma-Aldrich) and 0.5 ml ascorbic-acid solution (Sigma-Aldrich) in 3.9 ml deionized water were added, mixed thoroughly, and heated for 7 minutes in a boiling water bath. The optical density at 820 nm was recorded with a spectrophotometer (Ultraspec 2000, Pharmacia Biotec, Cambridge, UK).

### Tissue-mimicking phantom

The tissue-mimicking phantoms used in this study were constructed from an acrylamide/bis gel according to a previous report [13]. Briefly, the phantom was prepared from a polyacrylamide gel mixed with egg white (Sigma-Aldrich) as a hyperthermia indicator. The tissue-mimicking phantom consists of 10% acrylamide (Biosesang, Seomgnam, Korea), 30% egg white, 0.05% ammonium peroxodisulfate (Sigma-Aldrich), 4.5% glycerol, and 0.2% TEMED (Biosesang) in degassed water with or without Gd-C_5_F_12_-PLNs ([P] = 1 mM). The solution was then polymerized in an acrylic mold (Φ = 36 mm, height = 17 mm) to produce a cake of tissue-mimicking phantom.

### High-intensity focused ultrasound system and in vitro HIFU ablation

The equipment used to generate high intensity focused ultrasound is a commercially available device (TIPS, Therapy Imaging Probe System, Philips Research, Briarcliff Manor, NY) designed for preclinical research. , HIFU was applied to the tissue-mimicking phantom including Gd-C_5_F_12_-PLNs with an acoustic intensity of 5000 W/cm^2^ at the focal zone (6 dB) which is 1×1×6 mm^3^ and a sonication frequency of 1.0 MHz for 30 sec (duty cycle 100%). The effects of HIFU on the phantom were recorded with a CCD-camera and quantified by the brightness of the white opaque lesion using public domain software (Image J program, developed at the National Institutes of Health and available at http://rsb.info.nih.gov/nih-image/).

### Circulation time and distribution analysis of the Gd-C_5_F_12_-PLNs in mice

To determine circulation time, Gd-C_5_F_12_-PLNs (10 µmoles of P in 0.25 ml) were injected into BABL/c mice (Orient, Seongnam, Korea) via the tail vein. Blood was collected from the tail vein at various time points (30 min to 24 h) after injection. The distribution of Gd-C_5_F_12_-PLN in various organs was measured 24 hours after injection. Animals were sacrificed, and the muscle, kidney, liver, and spleen were harvested. The organs were then dried at 37°C for 48 hours. Gd quantities in the blood and organs were analyzed by Inductively Coupled Plasma-Atomic Emission Spectrometer (ICP-AES, Agilent 7500A, Palo Alto, CA).

To examine accumulation of the Gd-C_5_F_12_-PLN in the tumor tissue, the mouse colon cancer cell line CT-26 (5×10^6^ in 0.3 ml PBS) acquired from KCLB (Seoul, Korea) was subcutaneously injected into the left hind limbs of BABL/c nude mice. The tumor was allowed to grow for 10 days, reaching ∼10 mm in diameter. Mice were injected with Gd-C_5_F_12_-PLN-50 or Gd-C_5_F_12_-PLN-100 (10 µmoles of P in 0.25 ml) through the tail vein. Gd-C_5_F_12_-PLN accumulation in the tumor tissue was monitored with T1-weighted imaging (fast spin echo, TR/TE = 350/8.5 ms, slice thickness 1.0 mm) for 8 hours using a 7-Tesla small animal MR scanner (BioSpin70/20USR, Bruker-Biospin, Fallanden, Switzerland).

### In vivo HIFU ablation and MR assessment

The CT-26 tumor-bearing mice were injected with saline or Gd-C_5_F_12_-PLN-100 (0.4 mmoles of P/kg or 0.04 mmoles Gd/kg in 0.25 ml) through the tail vein. The tumor was treated by HIFU using TIPS. Each animal was anesthetized with isoflurane and placed in a custom-designed holder. The HIFU transducer was focused on the medial plane of the tumor and coupled onto the tumor with ultrasonic gel. HIFU was performed with ultrasound output power adjusted in the range of 3000 W/cm^2^, a frequency of 1.3 MHz, and a 50% duty cycle for 30 sec to ablate 2×2 mm^2^-area of tumor tissues. Tumor ablation was monitored by T1-weighted MR imaging (T1WI), before and 24 hours after HIFU treatment, performed in TR/TE = 8.5/350 ms with 1.0 mm slice thickness before and after injection of Dotarem (Guebet, Roissy, France) with 0.1 mmoles Gd/kg through the tail vein. To measure the area of ablated tumor tissue, each T1W image before Dotarem injection was subtracted from the corresponding image after injection. Animals were euthanized by carbon dioxide asphyxiation after imaging and the tumor tissue was collected for histological analysis. This study was reviewed and approved by the Institutional Animal Care and Use Committee (Approval ID: P-A9-502-2) of Samsung Biomedical Research Institute (SBRI) which is accredited by the Association for Assessment and Accreditation of Laboratory Animal Care International (AAALAC International) and abides by the Institute of Laboratory Animal Resources (ILAR) guide.

### Histopathological analysis

All frozen specimens were processed by both H&E and nicotinamide adenine dinucleotide reduced (NADH) diaphorase staining [14]. The specimens were cut to 4 µm thick and air-dried briefly at room temperature. The tissue sections were incubated at 37°C for 1 hour in 10 ml of Tris-HCl buffer (pH 7.4) containing 10 mg of nitro blue tetrazolium (Sigma-Aldrich) and 10 mg of NADH (Sigma-Aldrich). Specimens were washed with distilled water, various concentrations of acetone (30%, 60%, 90%, 60%, and 30%), and finally with distilled water. The specimens were mounted for later review by a pathologist. H&E staining was also performed on both frozen and paraffin-imbedded specimens using the standard technique. The ablated area seen by H&E staining was measured [length×width (mm^2^)] from tissue sections exposed perpendicular to the direction of HIFU propagation. The effect of Gd-C_5_F_12_-PLN-100 on the ablation area by HIFU (n = 6) was compared with Student's *t*-tests.

## Results

### Characterization of Gd-C_5_F_12_-PLN

**Figure 1:**
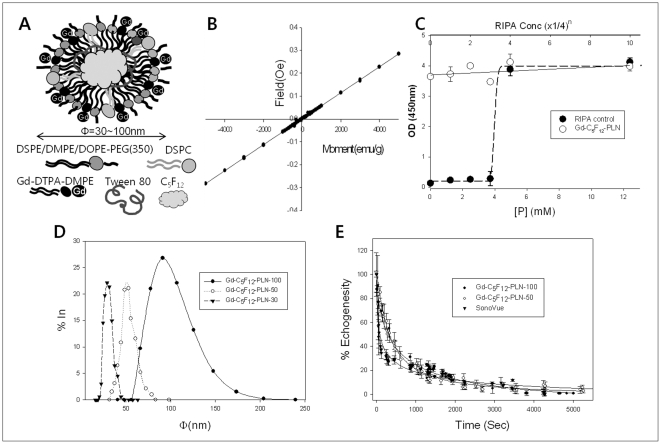
Chemical characterization of Gd-C_5_F_12_-PLNs. (A) Design of Gd-C_5_F_12_-PLN. Gd-C_5_F_12_-PLN was Φ = 30, 50, and 100 nm. (B) Paramagnetism of the Gd-C_5_F_12_-PLN from SQUID test. (C) Cytotoxicity of Gd-C_5_F_12_-PLNs in MS-1 cells. The cytotoxicity of Gd-C_5_F_12_-PLN was investigated by MTT assay. Serially diluted cell lysis buffer (RIPA) was used as a positive control. (D) Size-distribution of Gd-C_5_F_12_-PLNs from DLS analysis. (E) In vitro stability of Gd-C_5_F_12_-PLN-50, Gd-C_5_F_12_-PLN −100 and SonoVue was calculated from its echogenesity using high-frequency ultrasound.

The Gd-C_5_F_12_-PLN consists of a biocompatible phospholipid backbone, perfluoropentane gas, and Gd-DTPA (Fig. 1A). Various sizes of Gd-C_5_F_12_-PLN (Φ = 30, 50, 100 nm) were synthesized and its size distribution was confirmed by DLS (Fig. 1D). Gd and P contents in the Gd-C_5_F_12_-PLNs determined by ICP-AES and phosphorus assay, respectively were listed in Table 1. The Gd-C_5_F_12_-PLNs contained ∼0.1 mole Gd/mole P, meaning ∼20% of the DMPE-DTPA chelated Gd. The paramagnetic property of the Gd-C_5_F_12_-PLN confirmed with a SQUID test showed that the Gd-C_5_F_12_-PLN exhibits typical paramagnetism (Fig. 1B). The cytotoxicity of the Gd-C_5_F_12_-PLN in the tested cells was not found in the tested concentration range (Fig. 1C). In vitro bubble stability of Gd-C_5_F_12_-PLN-500, Gd-C_5_F_12_-PLN-100 at 37°C was significantly higher than that of SonoVue (Fig. 1E). IC_50_ of Gd-C_5_F_12_-PLN-50, Gd-C_5_F_12_-PLN-100 and SonoVue was 279.2±19.53, 310.2±20.53 and 90±12.24 sec, respectively.

**Table 1:** Chemical analysis of the synthesized phospholipid nanoparticles.

	[Gd] (mM)	[P] (mM)	[Gd]/[P]
Gd–PLN-30	**2.43**	**28.34**	**0.09**
Gd–PLN-50	**4.39**	**36.57**	**0.12**
Gd–PLN-100	**4.24**	**39.70**	**0.11**
Gd-C_5_F_12_–PLN-30	**3.97**	**37.13**	**0.11**
Gd-C_5_F_12_–PLN-50	**4.14**	**38.34**	**0.11**
Gd-C_5_F_12_–PLN-100	**3.80**	**43.88**	**0.09**

### Enhanced hyperthermal effect of HIFU by Gd-C_5_F_12_-PLN in vitro

**Figure 2:**
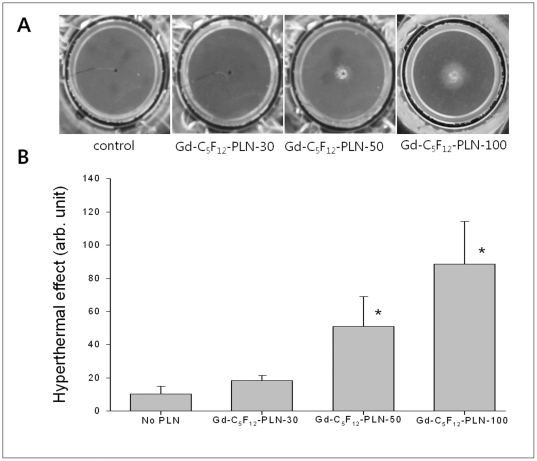
Effect of Gd-C_5_F_12_-PLN on the hyperthermal property of HIFU in the tissue-mimicking gel phantom. The phantom was monitored with a video camera while it was sonified for 1 min. The phantom was photographed after HIFU (A). The intensity of white opaque lesion in the phantom was measured as an indication of the hyperthermal effect (B). *P<0.05 (n = 5), Gd-C_5_F_12_-PLN-50 and Gd-C_5_F_12_-PLN-100 significantly increased the hyperthermal effect of HIFU compared to HIFU alone (no PLN).

To examine the effect of Gd-C_5_F_12_-PLN size on the hyperthermal property of HIFU, the polyacrylamide phantom was polymerized in the presence of three different Gd-C_5_F_12_-PLNs in diameter (30, 50, and 100 nm). HIFU without Gd-C_5_F_12_-PLNs did not show a significant turbidity change in the egg white of the transparent gel phantom (Fig. 2). Gd-C_5_F_12_-PLN enhanced the hyperthermal effect of HIFU in a size-dependent manner (Φ = 30<50<100 nm) (Fig. 2A), as determined by the turbidity of the white opaque lesion induced by HIFU (Fig. 2B). Both Gd-C_5_F_12_-PLN-50 and Gd-C_5_F_12_-PLN-100 significantly increased the hyperthermal effect of HIFU ablation, while Gd-C_5_F_12_-PLN-30 had only a slight effect. The video files recorded during HIFU exposure on the phantoms in the absence or presence of Gd-C_5_F_12_-PLN-30, Gd-C_5_F_12_-PLN-50 and Gd-C_5_F_12_-PLN-100 were shown in Supporting Information (Video S1, S2, S3, and S4, respectively).

**Figure 3:**
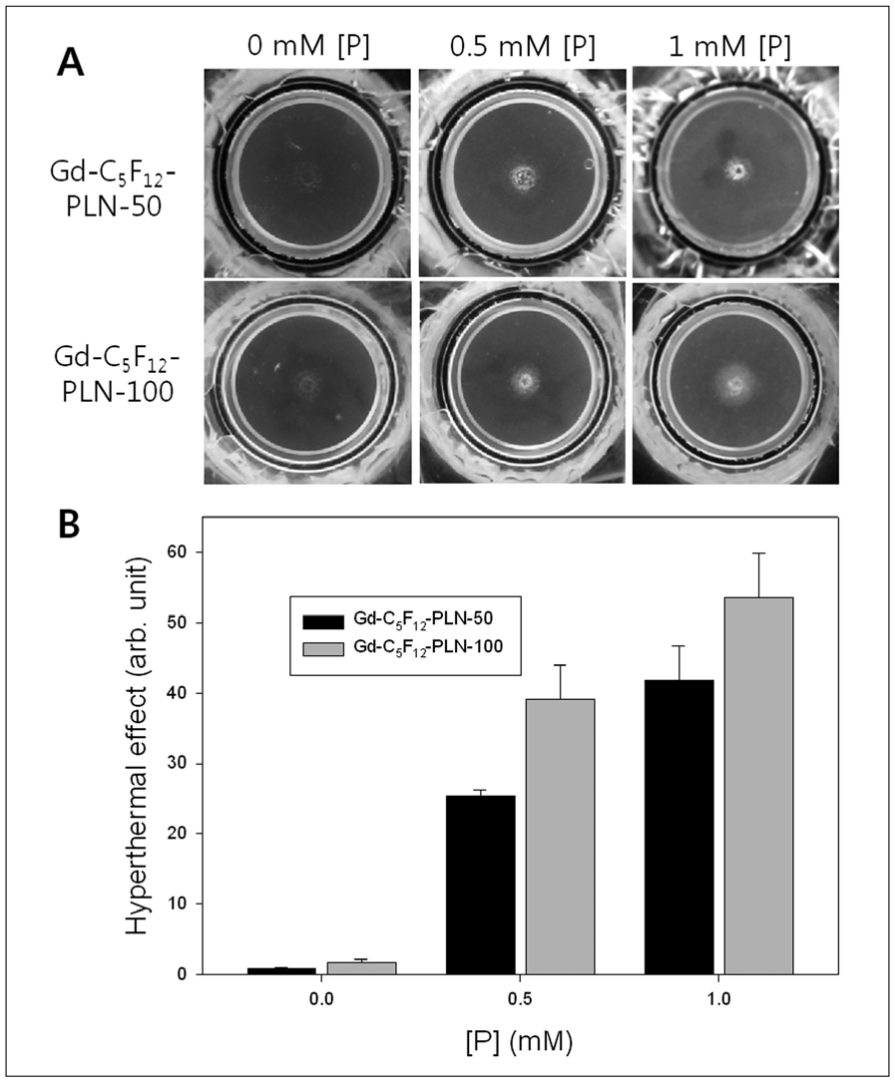
Concentration-dependent effect of Gd-C_5_F_12_-PLN on the hyperthermal property of HIFU in the tissue-mimicking gel phantom. The effect of Gd-C_5_F_12_-PLN-50 and Gd-C_5_F_12_-PLN-100 at different concentrations on the hyperthermal property of HIFU was examined in the phantom (A); the effect was quantified by the intensity of the opaque lesion (B). The Gd-C_5_F_12_-PLN concentration was determined by the phospholipid concentration, which is the main component of Gd-C_5_F_12_-PLN.

The concentration-dependent effect of both Gd-C_5_F_12_-PLN-50 and Gd-C_5_F_12_-PLN-100 on the hyperthermal property of HIFU was also observed (Fig. 3A). The intensity of HIFU lesions show that both Gd-C_5_F_12_-PLN-50 and Gd-C_5_F_12_-PLN-100 enhanced HIFU in a concentration-dependent manner (Fig. 3B).

### Circulation time and body distribution of Gd-C_5_F_12_-PLN in mice

**Figure 4:**
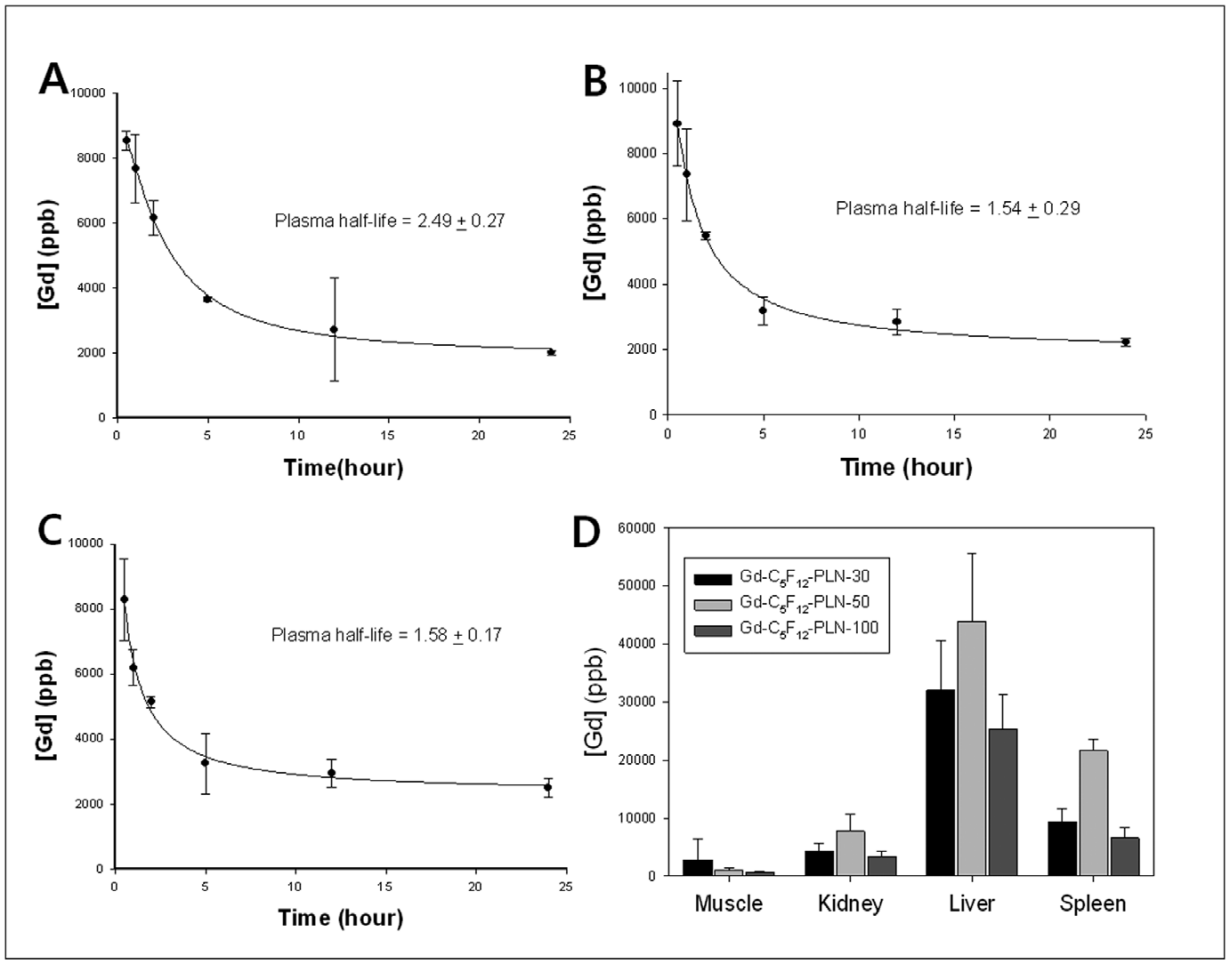
Circulation and distribution of Gd-C_5_F_12_-PLNs in mice. ICP-AES analysis of Gd in the blood after tail vein injection of the Gd-C_5_F_12_-PLNs showed that plasma half-life of Gd-C_5_F_12_-PLN-30 was 2.49±1.27 (A), Gd-C_5_F_12_-PLN-50 was 1.54±0.29 (B), and Gd-C_5_F_12_-PLN-100 was 1.58±0.17 (C). The body distribution of Gd-C_5_F_12_-PLNs was determined by [Gd] quantified by ICP-AES 24 hours after administering Gd-C_5_F_12_-PLNs in mice (D).

From the circulation times of Gd-C_5_F_12_-PLN-30, Gd-C_5_F_12_-PLN-50, and Gd-C_5_F_12_-PLN-100 measured by ICP-AES analysis of Gd, the plasma half-life of Gd-C_5_F_12_-PLN-30 (Fig. 4A), Gd-C_5_F_12_-PLN-50 (Fig. 4B), and Gd-C_5_F_12_-PLN-100 (Fig. 4C) was 2.49±0.27 hr, 1.54±0.29 hr, 1.58±0.17 hr, respectively.

The distribution of Gd-C_5_F_12_-PLN throughout the body was also examined to determine the pharmacokinetics and long-term clearance pathway (Fig. 4D). All Gd-C_5_F_12_-PLNs mainly accumulated in the liver and spleen 24 hrs after injection. These results imply its removal through the reticuloendothelial system (RES) in the liver and the spleen, resulting in slow secretion of Gd-C_5_F_12_-PLNs through the hepatobiliary pathway.

### Accumulation of Gd-C_5_F_12_-PLNs in tumor tissues

**Figure 5:**
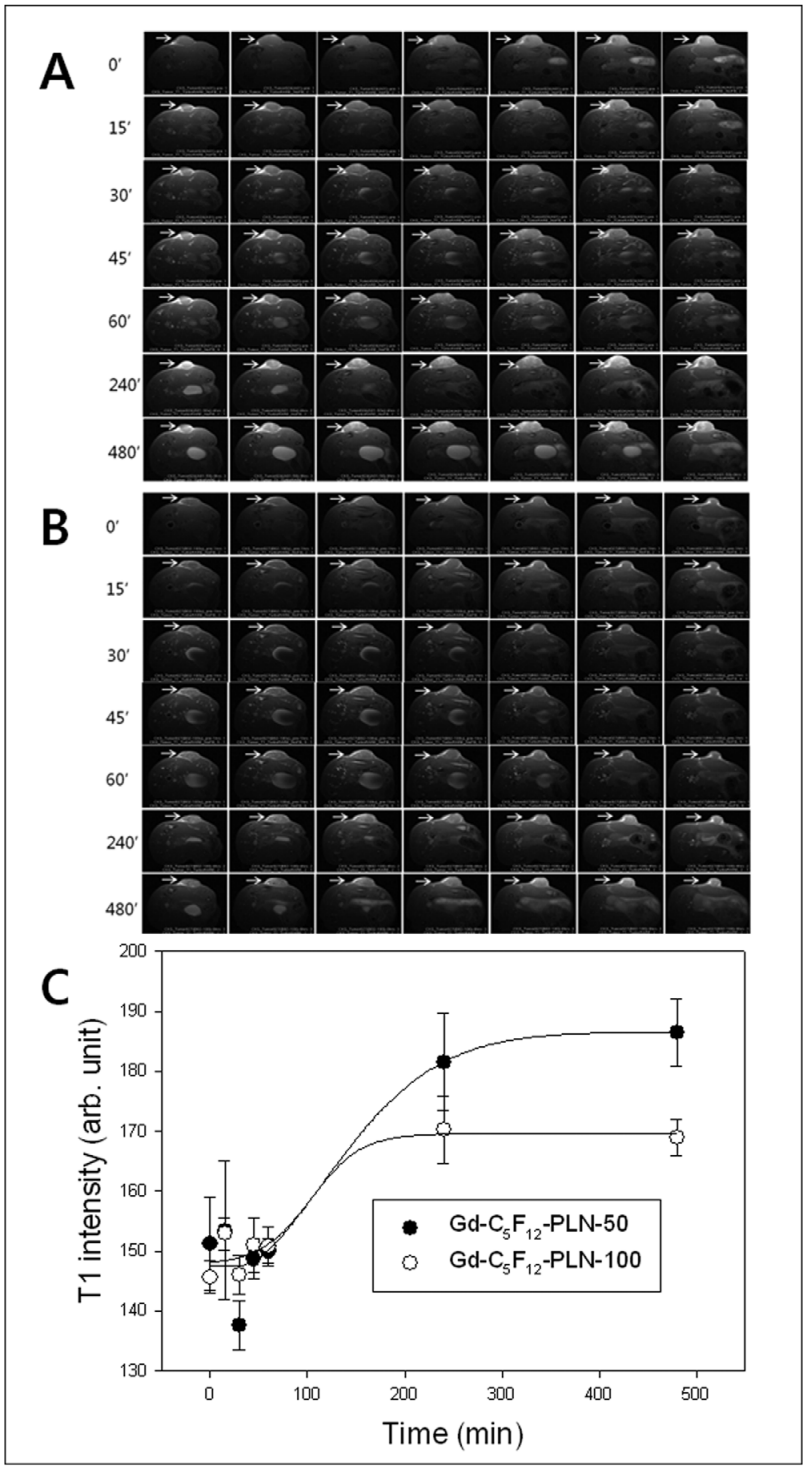
Accumulation of Gd-C_5_F_12_-PLNs in tumor tissues in mice. The tumor tissues were monitored with T1-weighted imaging for 8 hours after injection of Gd-C_5_F_12_-PLN-50 (A) and the Gd-C_5_F_12_-PLNs-100 (B). The changes of T1 intensity were plotted in C.

After intravenous injection of Gd-C_5_F_12_-PLN-50 (Fig. 5A) or Gd-C_5_F_12_-PLN-100 (Fig. 5B), the time-dependent accumulations of both Gd-C_5_F_12_-PLN-50 and Gd-C_5_F_12_-PLN-100 in the tumor tissue monitored with T1-weighted MR imaging were observed and plotted, as shown in Figure 5C.

### Enhancement of HIFU ablation by Gd-C_5_F_12_-PLN in tumor-bearing mice

**Figure 6:**
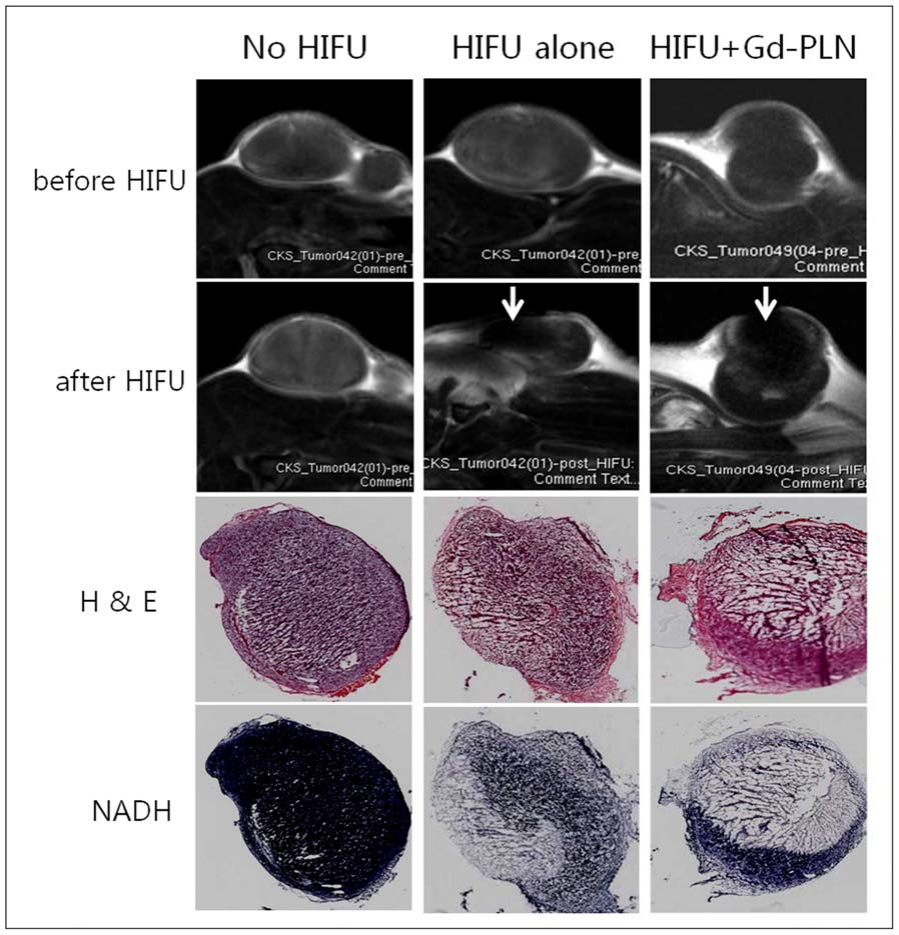
The effect of the Gd-C_5_F_12_-PLN-100 on HIFU tumor ablation in the subcutaneous tumor model. The tumor ablation by HIFU was monitored by gadolinium -enhanced T1-weighted imaging before and after HIFU treatment. The contrast-enhanced images were subtracted from the image before enhancement. The arrow indicates the HIFU orientation (upper panel). The HIFU ablation was also examined by H&E and NADH staining of frozen specimens to confirm the ablated area (lower panel).

**Figure 7:**
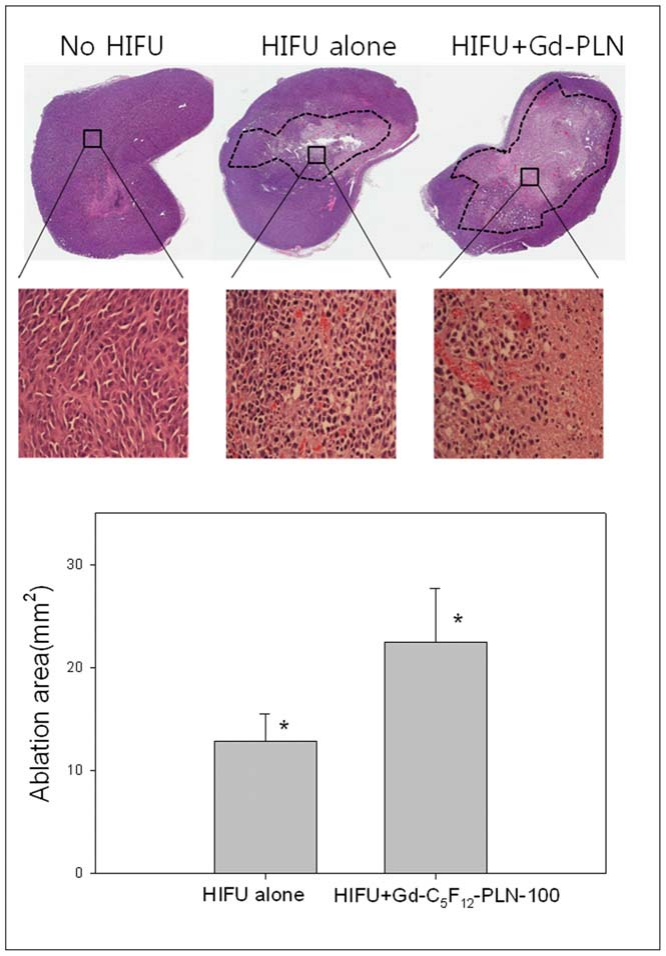
H&E analysis of tumor tissues treated with HIFU in the presence or absence of Gd-C_5_F_12_-PLN-100. The staining was performed on paraffin-imbedded specimens (upper panel). The dotted area represents the ablated tumor tissue. The ablated area perpendicular to the HIFU direction was measured and shown with histogram (lower panel). *P<0.025, the Gd-C_5_F_12_-PLN-100 significantly increased the HIFU-induced tumor ablation (n = 6).

To examine the effect of Gd-C_5_F_12_-PLN on hyperthermal tumor cell ablation of HIFU, HIFU was applied to a subcutaneous tumor model in mice injected with Gd-C_5_F_12_-PLN-100 or vehicle (Fig. 6). The ablated region in T1WI and H&E staining corresponded with the dead cell area denoted by NADH staining. The tumor tissues after sonication was also examined in a paraffin-imbedded specimen to cytologically confirm that the ablated area contained few abnormal cells (Fig. 7). HIFU treatment with Gd-C_5_F_12_-PLN-100 ablated a significantly larger area than no HIFU or HIFU alone (*p* = <0.025, n = 6) (Fig. 7, lower panel).

## Discussion

Although microbubble contrast agents are known to enhance the therapeutic effect of HIFU ablation [15], [16], no contrast agents are being used clinically because of its short plasma half-life. We thus designed and prepared gas-filled nanobubbles (Φ = 30–100 nm) that are smaller than microbubble contrast agents (Φ = 1–5 µm) to increase their stability (Fig. 1A). Even though the stability of nanobubbles is still controversial because of its very high Laplace pressure resulting in quick dissolution and rapid disappearance [17], [18], it has been reported that nanobubbles can form freely and remain stable for long periods of time due to a lower interfacial curvature with a high contact angle resulting in stability of nanobubble by reduction of the Laplace pressure [19]–[21]. Our in vitro stability data showed that IC_50_ of Gd-C_5_F_12_-PLN-50 and 100 was three times longer than that of SonoVue (Fig. 1E).Our nanobubbles mainly composed of biocompatible phospholipids were infused with decafluoropentane gas, resulting that Gd-C_5_F_12_-PLN-50 and Gd-C_5_F_12_-PLN-100 enhanced the hyperthermal property of HIFU in both a size- and concentration-dependent manner (Fig. 2 and 3). This enhancement of the hyperthermal effect could reduce the HIFU energy required to ablate a tumor, thus reducing the procedure time and the side effects of HIFU, such as skin burns.

Gd-C_5_F_12_-PLN seems cleared through biliary excretion through RES of liver and the spleen (Fig. 4D). The biliary clearance pathway of the Gd-C_5_F_12_-PLN suggests that the Gd-conjugated nanobubbles might not cause nephrogenic system fibrosis (NSF), a common complication of small molecular weight Gd chelates [22]. A Gd-conjugated lipid nanoparticle similar to our Gd-C_5_F_12_-PLN had little effect on nephrogenic system fibrosis [23]. In addition to the clearance pathway bypassing NSF, we only used 40% of the recommended clinical dose of Gd (0.1 mmoles/kg), suggesting that the Gd-C_5_F_12_-PLN might increase the margin of safety in treating patients.

As intended, the nano-sized Gd-C_5_F_12_-PLN remained in circulation long enough to be used in HIFU, comparable to microbubble contrast agents (Fig. 4). The plasma half-lives of Gd-C_5_F_12_-PLN-50 and Gd-C_5_F_12_-PLN-100 in mice were 1.54±0.29 hr and 1.58±0.17 hrs, respectively, which is likely longer than 5 hrs in humans based on the allometric scale between mice and humans [24]. Because Gd-C_5_F_12_-PLNs had a long circulation time, HIFU agents will maintain their therapeutic effects during treatment, which can take several hours to effectively ablate a large tumor. In addition, the long circulation time also provides a window for the agent to accumulate in tumor tissues that have highly permeable, angiogenic microvessels (*i.e.*, EPR). Gd-C_5_F_12_-PLN-50 and Gd-C_5_F_12_-PLN-100 monitored by T1WI continuously increased in the tumor tissues until at least 4 hrs after injection (Fig. 5). The tumor accumulation allows various treatment options in HIFU therapy. HIFU treatment can used after a bolus injection of the HIFU agent, or after it accumulates in tumor tissues by EPR. The effects of HIFU treatments with Gd-C_5_F_12_-PLNs should to be examined in detail for various stages and types of tumors to help design therapeutic plans for HIFU ablation. The accumulation of the Gd-C_5_F_12_-PLN-50 in tumor tissues is much higher than Gd-C_5_F_12_-PLN-100, probably due to their relative stabilities in vivo (Fig. 5C). Thus Gd-C_5_F_12_-PLN-50 may be useful for the delayed HIFU ablation after it accumulates in tumor tissues, although Gd-C_5_F_12_-PLN-100 has a greater hyperthermal effect (Fig. 2). Gd-C_5_F_12_-PLN-50 may also have potential advantages to design its tumor-specific delivery using a molecular targeting technique, which also requires a long circulation time.

Our gas-filled nanobubbles were conjugated with Gd-DTPA to be detectable by MRI. Gd-C_5_F_12_-PLN had paramagnetic properties by SQUID (Fig. 1B) and T1 enhancement in mice (Fig. 5). Ten percent of the phospholipids in Gd-C_5_F_12_-PLNs were conjugated with Gd through chelation with DMPE-DTPA (Table 1). MR imaging with the Gd-C_5_F_12_-PLN may provide various advantages, such as visualizing the HIFU agent in the tumor tissue to be ablated, diagnosing of hyper-vascular tumor tissues as a blood pool agent, and determining the HIFU treatment time by monitoring accumulation of the HIFU agent in the tumor.

Despite the many advantages of Gd-C_5_F_12_-PLNs in enhancing HIFU ablation, there are still issues to address before using Gd-C_5_F_12_-PLN clinically. First, inertial cavitation from combining HIFU and gas-filled Gd-C_5_F_12_-PLNs is unpredictable and cause complications. This risk has been a barrier to using microbubble agents clinically, as well. Because Gd-C_5_F_12_-PLN is much smaller than microbubbles, the potential complications of acoustic cavitation would likely be much smaller. Second, Gd-C_5_F_12_-PLNs were removed by RES and excreted via the hepatobiliary pathway, thus the risk of nephrogenic systemic fibrosis is likely lower than Gd-based MR contrast agents. However, the effect of HIFU-induced hyperthermia on Gd chelation of the nanobubbleshas not been well studied, therefore HIFU might release free gadolinium, although it might be minimal. Once these issues are solved, Gd-C_5_F_12_-PLN has a great deal of potential in clinical HIFU therapy.

Taken together, in vitro and in vivo data demonstrated that Gd-C_5_F_12_-PLN, which is stable and detectable by MR imaging, is a potential theragnostics for the MR-guided HIFU ablation. Even though we only assessed the role of Gd-C_5_F_12_-PLN in enhancing the hyperthermal effect of HIFU therapy, we are convinced it has many other applications for focused ultrasound, such as ultrasound-assisted drug/gene delivery or blood-brain barrier opening, which should be characterized in future studies.

## Supporting Information

Video S1 **Hyperthermal effect of HIFU on a acrylamide gel phantom in the absence of Gd-C_5_F_12_-PLN.** The real-time video recording was taken during the whole HIFU exposure time.
(WMV)

Video S2 **Hyperthermal effect of HIFU on a acrylamide gel phantom containing Gd-C_5_F_12_-PLN-30.** The real-time video recording was taken during the whole HIFU exposure time.
(WMV)

Video S3 **Hyperthermal effect of HIFU on a acrylamide gel phantom containing Gd-C_5_F_12_-PLN-50.** The real-time video recording was taken during the whole HIFU exposure time.
(WMV)

Video S4 **Hyperthermal effect of HIFU on a acrylamide gel phantom containing Gd-C_5_F_12_-PLN-100.** The real-time video recording was taken during the whole HIFU exposure time.
(WMV)
